# Extracellular vesicles carrying miRNA-181b-5p affects the malignant progression of acute lymphoblastic leukemia

**DOI:** 10.1186/s12967-021-03174-w

**Published:** 2021-12-18

**Authors:** Wei Yan, Li Song, Huihan Wang, Wei Yang, Liang Hu, Ying Yang

**Affiliations:** 1 Department of Hematology, Shengjing Hospital of China Medical University, Shenyang, 110000 People’s Republic of China; 2 National Drug Clinical Trial Institute Office, Qingdao Women and Children’s Hospital, Qingdao, China; 3 Shanghai Engineering Research Center of Pharmaceutical Translation, Shanghai, China

**Keywords:** miRNA-181b-5p, ALL, Extracellular vesicles, Proliferation, Cell cycle

## Abstract

**Objective:**

To investigate how serum extracellular vesicles (EVs)-carried miRNA-181b-5p affected the proliferation, cell cycle and apoptosis of acute lymphoblastic leukemia (ALL) cells.

**Methods:**

Differentially expressed miRNAs related to ALL were screened by bioinformatics analysis, and the localization of target miRNA was searched by its expression. qRT-PCR was adopted to confirm the expression of miRNA-181b-5p. Flow cytometry and fluorescence microscopy were applied to evaluate EVs internalization. MTT assay was employed to verify the proliferation of ALL cells. Cell cycle and apoptosis were analyzed by flow cytometry. Transwell assay was applied to evaluate migration and invasion abilities.

**Results:**

High expression of miRNA-181b-5p was proved in ALL cell lines, and miRNA-181b-5p enriched in the exosomes and vesicles of blood cells. In the meantime, it was found that EVs carrying miRNA-181b-5p could be internalized by ALL cells and thus the expression of miRNA-181b-5p was up-regulated. Cell function assays showed that the proliferation, migration, invasion abilities of ALL cell lines were promoted in miRNA-181b-5p mimic group or the group co-culturing ALL-derived EVs and BALL-1 cell lines. The percentage of cells in G0/G1 phase was reduced and cell apoptosis was also inhibited.

**Conclusion:**

miRNA-181b-5p carried by EVs in peripheral blood of ALL patients can enter ALL cells and thus promote the malignancy of ALL cells.

## Introduction

Acute lymphoblastic leukemia (ALL) is a kind of tumor derived from the abnormal proliferation of B-lymphoblasts or T-lymphoblasts in the bone marrow. Due to the fast onset, patients with ALL, if not treated in time, can only survive for from a few weeks to several months. However, the overall survival (OS) rate of adult patients who underwent allogeneic hematopoietic stem cell transplantation could be increased from 15–45% to 27–65% [1, 2]. ALL accounts for about 20% of adult leukemia cases while the disease is more prevalent in childhood (0–9 years old), accounting for more than 70% of childhood leukemia [3]. From the current treatment of ALL, the cure rate of child patients with ALL is close to 90% [4], but the treatment for adult patients with ALL is still facing great challenges [5, 6].

Recently, extracellular vesicles (EVs) have attracted much attention in cancers because of the potential of EVs as unique biomarkers or intercellular mediators, and functions against metastasis and recurrence [7, 8]. Exosomes and micro-vesicles (MVs) are two major subtypes of EVs [9, 10] that are produced by healthy cells or cancer cells and can be detected in multiple body fluids, such as blood, urine or saliva [8]. EVs can deliver bioactive molecules from one cell to another, like functional RNA and proteins [11, 12], and membrane EVs are carriers delivering information to receptor cells [13]. Several studies reported that EVs derived from cancer cells relate to microRNA (miRNA) enrichment in biological fluids of patients with cancers, suggesting that miRNA may be a marker to predict the prognosis of cancer [14, 15]. Li et al. found that 182 miRNAs and 166 miRNAs were differentially expressed in Nalm-6-MVs and Jurkat-MVs respectively, and these abnormally expressed miRNAs target a variety of oncogenes, tumor suppressor genes and signaling pathway genes, which may be involved in the progression of B-ALL or T-ALL [16]. Nevertheless, works are still less focused on the delivery of miRNAs from EVs into ALL cells and on their effects in the procession of ALL.

Herein, the expression level of miRNA-181b-5p in serum EVs from patients with ALL or healthy people was evaluated. miRNA-181b-5p was proved remarkably up-regulated in EVs derived from ALL patients, and it might be a regulator in ALL cells.

## Materials and methods

### Bioinformatics analysis

Expression microarray GSE56489 of ALL related miRNA was obtained from GEO database (https://www.ncbi.nlm.nih.gov/geo/). R “limma” package was used to perform differential analysis, with the control of normal samples, and |logFC|> 1.5 along with adj.pvalue < 0.05 was used as the screening standard of differentially expressed miRNAs (DEmiRNAs). On the EVmiRNA database (http://bioinfo.life.hust.edu.cn/EVmiRNA#!/), the expression of the target miRNA was localized.

### Peripheral blood collection and serum separation

Peripheral blood samples were obtained from 14 ALL patients and 14 healthy individuals with vacuum SST II tubes (Becton Dickinson, BD, Franklin, NJ, USA), and the serum was collected after the centrifugation of samples at 1100×*g* for 20 min. All serum samples were stored at − 80 ℃ for use.

### Cell line and culture

ALL cell line BALL-1 (BNCC102176) was procured from BeNa Culture Collection. The cell line was cultivated in a moist incubator containing 5% CO_2_ at controlled temperature of 37 ℃. The culture medium was RPMI-1640 (HyClone, Logan, UT, USA) with 10% fetal bovine serum (FBS) supplement (Gibco, Grand Island, NY, USA).

### Cell transfection

miRNA-181b-5p mimic, mimic NC, miRNA-181b-5p inhibitor, inhibitor NC were acquired from GenePharma (Shanghai, China). BALL-1 cells were seeded in a 6-well plate and transfected with mimics adopting the Lipofectamine 2000 reagent (Invitrogen, Carlsbad, CA, USA). After 48 h, the cells were gathered for subsequent assays.

### RNA extraction and qRT-PCR assay

TRIzol reagent (Invitrogen, Carlsbad, CA, USA) was employed to extract total RNA from human peripheral blood samples and cancer cells. As for quantifying the expression of miRNA-181b-5p, complementary DNA (cDNA) was synthesized by the miScript reverse transcription kit (Qiagen), and qRT-PCR assay was conducted with the SYBR Premix Ex Taq (TaKaRa, Otsu, Shiga, Japan). U6 was the internal reference of miRNA-181b-5p. 2^−ΔΔCt^ value was used for data quantification and normalization. The qRT-PCR was performed by the Applied Biosystems 7500 system (Applied Biosystems, Warrington, UK). Primers adopted were: miRNA-181b-5p: forward primer 5′-CCAGCTGGGCTCACTGAACAATGA-3′ and reverse primer 5′-CAACTGGTGTCGTGGAGTCGGC-3′; U6: forward primer 5′-GCTTCGGCAGCACATATACTAAAAT-3′ and reverse primer 5′-CGCTTCAGAATTTGCGTGTCAT-3′.

### Western blot

After the transfection, the cells in each group were washed with cold phosphate-buffered saline (PBS) 3 times, and then cell lysis was conducted on ice for 10 min after cell lysis buffer was added. The concentration of protein was detected by BCA kit (Thermo Fisher Scientific, Waltham, MA, USA). SDS–Polyacrylamide gel electrophoresis was carried out for 30 μg of the total proteins. After electrophoresis, the proteins were removed to a PVDF membrane (Amersham, USA). 5% skim milk powder was added to block the membrane at room temperature for 1 h. After discarding the blocking solution, CD63 (ab59479, 1:10,000, abcam, Cambridge, UK), CD9 (ab92726, 1:2000, abcam, Cambridge, UK), TSG101 (ab125011, 1:2000, abcam, Cambridge, UK), CD19 (ab134114, 1:2000, abcam, Cambridge, UK), CD40 (ab224639, 1:1000, abcam, Cambridge, UK) and GAPDH (ab9485, 1:2500, abcam, Cambridge, UK) were added to the membrane for incubation at 4 °C overnight. All of the primary antibodies were rabbit polyclonal antibodies. Then, PBST (PBS buffer containing 0.1% Tween-20) was subject to the membrane 3 times, 10 min at a time. After that, horseradish peroxidase-labeled secondary Goat anti-rabbit IgG H&L (ab6721, 1:2000, abcam, Cambridge, UK) was added and incubated at room temperature for 1 h. PBST was again applied to wash the membrane 3 times, 10 min at a time. After the protein being scanned and developed, the protein signals were photographed with the optical luminometer (GE, USA).

### MTT assay

MTT assay was applied to measure the proliferation of ALL cells. The treated cell line (5 × 10^3^ cells/100 μl) was plated into a 96-well plate. After 24, 48 and 72 h, the cell proliferation was evaluated with sterile MTT solution (Beyotime). Spectrophotometer (Molecular Devices, Sunnyvale, CA, USA) was utilized to observe and record the optical density at 570 nm.

### Cell cycle analysis

The seeded concentration of cells in a 6-well plate was 5 × 10^4^ cells/ml, with about 2 ml of cell suspension. After 72 h, the cell suspension was centrifuged at 1000×*g* for 3 min for cell collection. Thereafter, the cells were kept overnight with 70% ethanol at 4 ℃ for fixation. The next day, after the centrifugation for cells, the supernatant was decanted. The cells acquired were subject to PBS for wash. Afterwards, the cells were dyed with 20 μg/ml propidium iodide (PI) solution (Sigma, USA) and 200 μg/ml RNase A (Sangon, China) at 37 ℃ for 30 min in the dark, and then cells underwent flow cytometry (BD Biosciences, USA). Pulse signals acquired from flow cytometry were evaluated by ModFit software (Verity Software House, Topsham, ME).

### Cell apoptosis assay

Cell apoptosis was measured by the Annexin V-PI fluorescein staining kits (Bender Medsystems, Austria). 5 × 10^4^ cells were suspended in 500 μl (1×) binding buffer (10 mM HEPES, pH = 7.4, 140 mM NaCl, 2.5 mM CaCl_2_). Then, the cells were blocked with Annexin V (1:20) for 5 min and PI was subsequently added for 15 min. Thereafter, cells were subject to flow cytometry.

### Extraction of EVs

The serum collected underwent centrifugation at 12,000×*g* at 10 ℃ for 20 min. Then it was moved into a 4 ml ultracentrifugation tube. Fixed angle rotor Ti 50.4 (Beckman Coulter) was used and the serum samples underwent centrifugation at 10,000×*g* at 10 ℃ for 70 min. Subsequently, the supernatant was decanted. EVs were resuspended by 2 ml PBS and then ultracentrifuged at 100,000×*g* at 10 ℃ for 70 min. The final EVs particles were suspended in 250 μl PBS.

### Evaluating EVs internalization

In order to evaluate the internalization of EVs, EVs obtained were revitalized in the buffer in kits from Sigma Aldrich (Germany), and PKH67 was also added for incubation at room temperature for 5 min. Afterwards, the samples were mixed with PBS with 5% FBS. The rest dye was removed by ultra-centrifugation at 100,000×*g* for 1 h. The stained EVs were co-cultivated with BALL-1 cells (stained by DAPI) for 6 h, and then subject to 4% paraformaldehyde at 25 ℃ for 20 min. The internalization of EVs was evaluated by a fluorescence microscope.

To furtherly examine the internalization, the co-cultured cells were analyzed using flow cytometry. The uptake rate was calculated following the method in the previous study (30087865).

### Transwell assay

Transwell assay was introduced to assess cell migration and invasion abilities using transwell chamber (Corning, USA). For migration assay, the transfected cells were seeded into the upper chamber (10^5^ cells/well) in the medium without suppling FBS. Meanwhile, the medium containing 10% FBS was added to the lower chamber. 24 h later, the cells on the upper chamber were removed using cotton swab, followed by quantifying migrated cells by MTT assay. For the invasion assay, the upper chamber was coated with Matrigel (BD, USA), and the remaining steps followed the steps in the migration assay.

### Statistical analysis

GraphPad Prism 7 software (GraphPad software, Inc., La Jolla, CA) was adopted to process data. The measurement data were manifested in the form of mean ± standard deviation. The comparison between groups was conducted by *t* test, while that among groups was subject to one-way ANOVA. *P* < 0.05 indicated a statistical significance.

## Results

### High expression of miRNA-181b in ALL

**Fig. 1:**
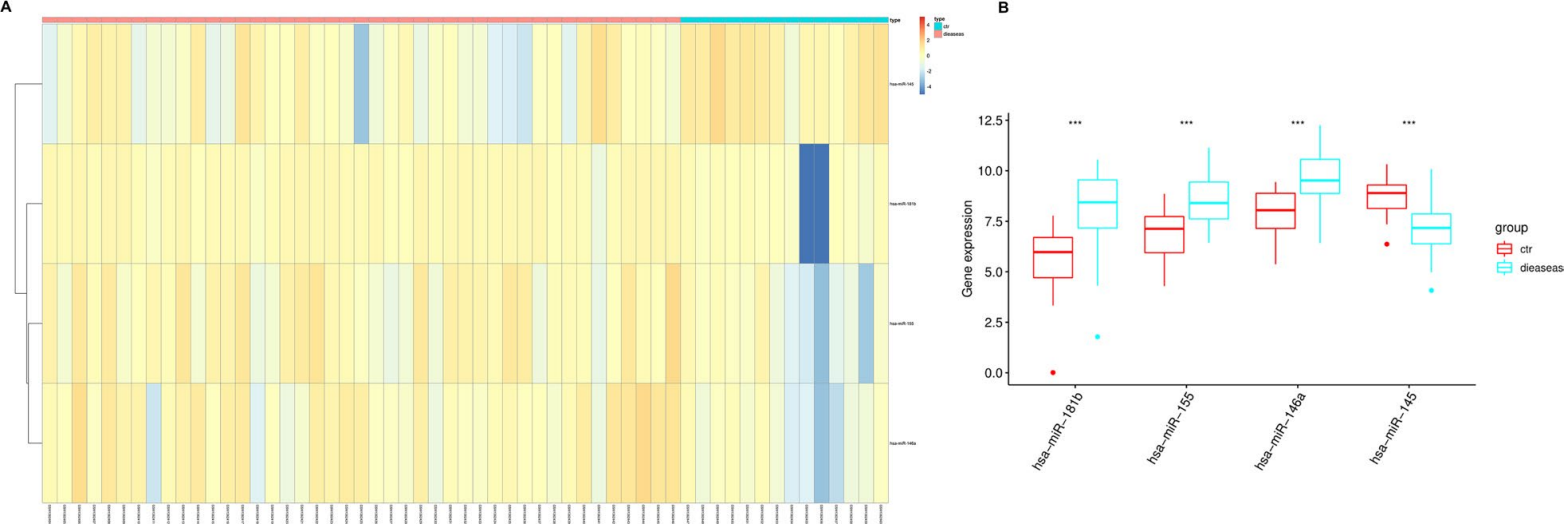
Highly-expressed miRNA-181b in ALL. **A** Heatmap of GSE56489 DEmiRNAs; **B** box plots show differential expression of 4 candidate miRNAs; ***p < 0.001

ALL-related miRNA expression chip GSE56489 (Fig. 1A) was acquired from GEO database, with 14 normal samples and 43 ALL samples. R package “limma” was utilized to acquire DEmiRNAs. |logFC|> 1.5 along with adj.pvalue < 0.05 were the screening standards for DEmiRNAs. In total, 4 DEmiRNAs were obtained, among which miRNA-181b, miRNA-155 and miRNA-146a were up-regulated and miRNA-145 was down-regulated (Fig. 1B). miRNA-181b expressed the most significantly. Therefore, miRNA-181b was chosen as the research object.

### miRNA-181b-5p regulates the proliferation, apoptosis and cycle of ALL cells

**Fig. 2:**
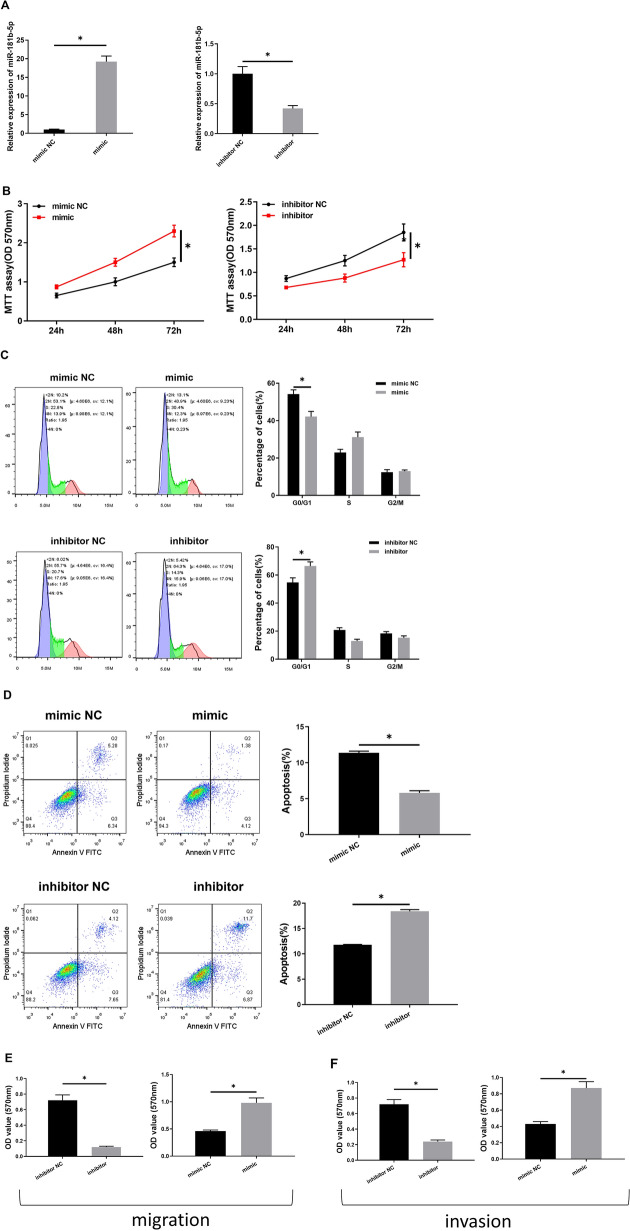
miRNA-181b-5p regulates the proliferation, apoptosis and cell cycle of ALL cells. **A** The transfection efficiency; **B** the proliferative activities of the cells; **C** the cell cycles of the cells; **D** the apoptotic rates of the cells; **E**, **F** migration and invasion of the cells; *p < 0.05

To furtherly understand the impacts of miRNA-181b-5p on the biological functions of ALL cells, miRNA-181b-5p mimic, mimic NC, miRNA-181b-5p inhibitor, and inhibitor NC were transfected with BALL-1 cells. The expression levels of miRNA-181b-5p in the transfected cells were examined to determine transfection efficiency (Fig. 2A). Then, the cell proliferation, apoptosis, cell cycle, migration, and invasion abilities were assessed, and the results revealed the significant effect of miRNA-181b-5p on the biological functions of ALL cells. As shown in Fig. 2B–F, in the miRNA-181b-5p mimic group, cells had higher proliferative ability than the cells in the NC group, with the proportion of cells in G0/G1 phase lowered, while the apoptotic rate decreased. Meanwhile, migration and invasion abilities of cells were strengthened. While the opposite effects were observed in the groups treated with miRNA-181b-5p inhibitor or inhibitor NC. The results indicated that the progression of ALL cells could be promoted after miRNA-181b-5p was overexpressed.

### EVs are abundant in peripheral blood of ALL patients and can be internalized by BALL-1 cells

**Fig. 3:**
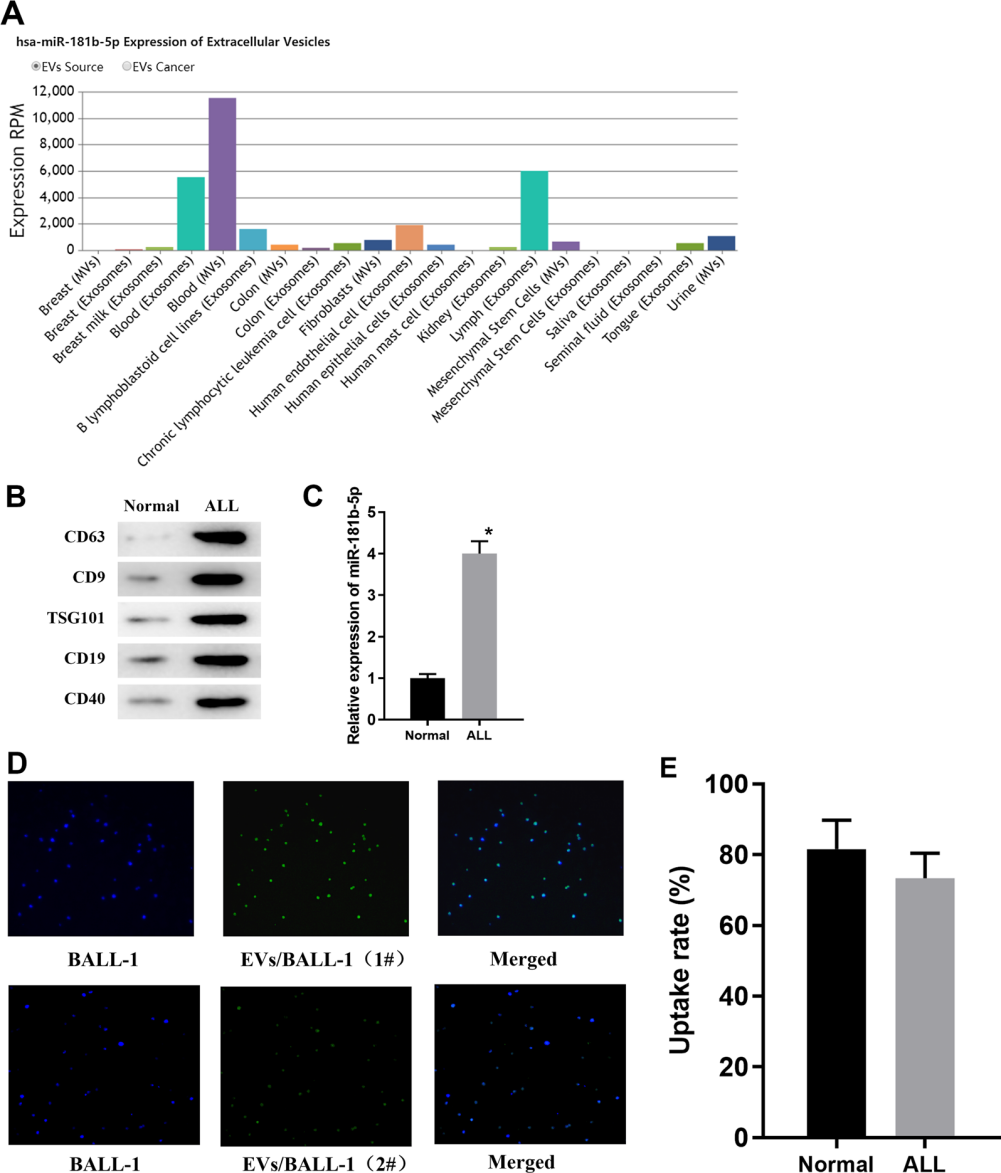
EVs are abundant in peripheral blood of ALL patients and can be internalized by BALL-1 cells. **A** Expression column diagram of miRNA-181b-5p in EVs from different tissues, with the X-axis representing cell type and the Y-axis representing expression RPM; **B** protein expression of surface markers of EVs from healthy people and ALL patients; **C** the expression level of miRNA-181b-5p in EVs from healthy people and ALL patients; **D** the internalization of EVs by BALL-1 cells; **E** the EVs uptake rate was calculated by flow cytometry; *p < 0.05

The distribution of miRNA-181b-5p was examined via EVmiRNA database, finding that miRNA-181b-5p is mainly presented in exosomes, MVs and exosomes of lymphocytes (Fig. 3A). Therefore, it was speculated whether EVs-derived miRNA-181-5p could enter ALL cells and thus plays a role. Therefore, EVs in peripheral blood samples from ALL patients and healthy people were obtained by ultracentrifugation. Surface marker proteins of EVs were detected via Western blot assay. As the results illustrated, the expression levels of proteins like CD63, CD9, TSG101, CD19 and CD40 were relatively intensive in EVs from ALL patients compared to the healthy group (Fig. 3B). This indicated that EVs from the peripheral blood samples of ALL patients and healthy people were successfully isolated. In addition, qRT-PCR assay unveiled that the expression of miRNA-181b-5p was significantly higher in EVs from patients than that in healthy controls (Fig. 3C). To verify whether miRNA-181b-5p in EVs can enter and affect the activity of ALL cells, EVs from patients were labeled with PKH67 fluorescence and it was found that they were effectively internalized by BALL-1 cells (Fig. 3D).Meanwhile flow cytometry results presented the uptake rate of EVs (Fig. 3E), indicating that EVs can be transferred from serum to the receptor cell BALL-1, and EVs-derived miRNA-181b-5p might enter ALL cells through internalization and thus affect the progression of ALL.

### miRNA-181b-5p in ALL-derived EVs impacts the proliferation, apoptosis and cell cycle of ALL cells

**Fig. 4:**
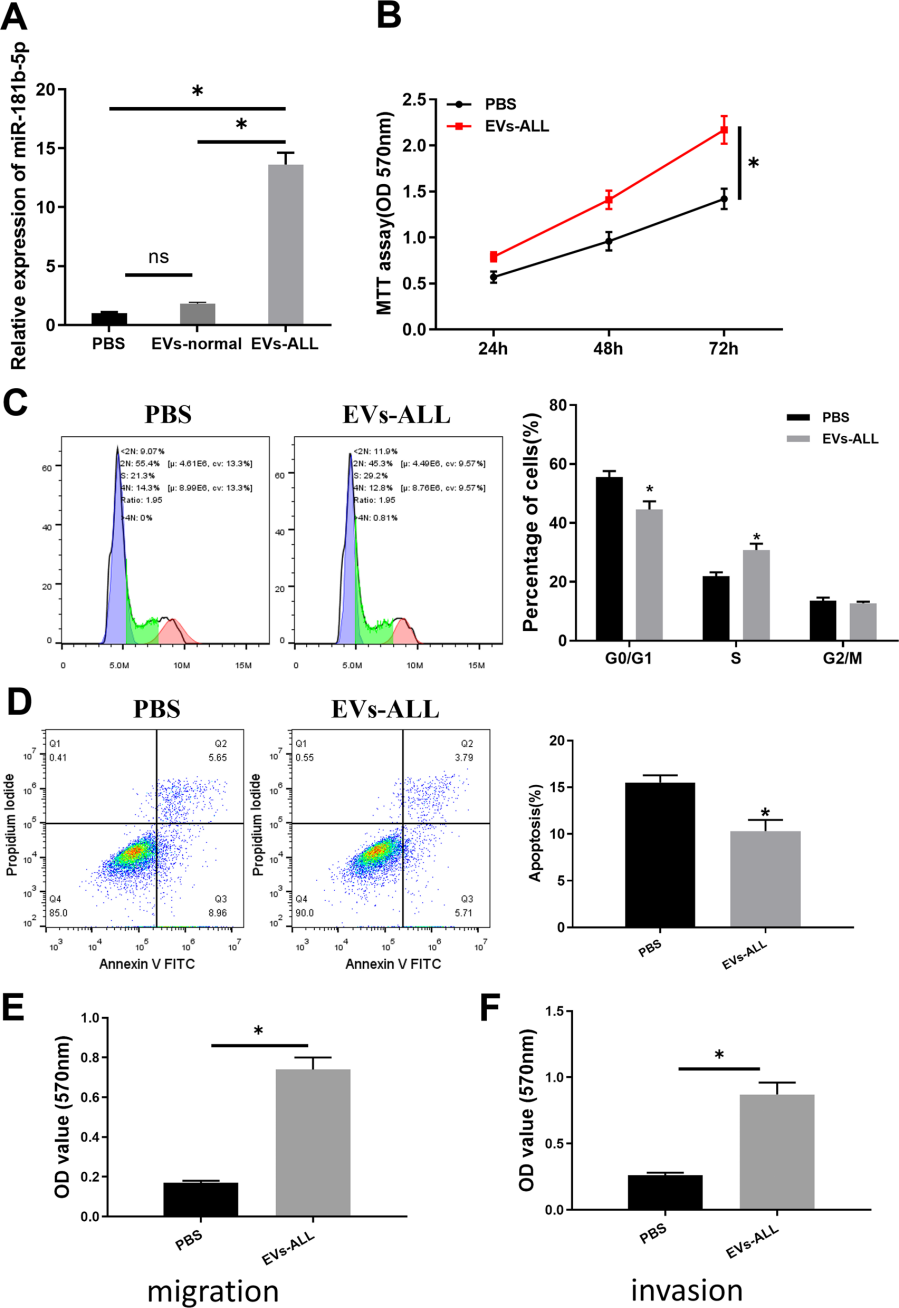
miRNA-181b-5p in EVs from ALL patients affects the proliferation, apoptosis and cell cycle of BALL-1 cells. **A** The expression of miRNA-181b-5p in each group of BALL-1 cells; **B** the viability of cells; **C** the cell cycle of each transfection group; **D** the cell apoptotic rate of each transfection group; **E**, **F** cell migration and invasion; *p < 0.05

To further explore the impact of EVs from peripheral blood of ALL patients on ALL cellular functions, BALL-1 cells were co-cultured with EVs derived from ALL and healthy people. The expression of miRNA-181b-5p in BALL-1 was remarkably up-regulated in the EVs-ALL group (Fig. 4A), which indicated that miRNA-181b-5p from ALL patients could enter BALL-1 cells with its expression improved. Then, MTT was employed to verify the viability of ALL cells in different groups. The results illustrated that the viability of ALL cells in EVs-ALL group was remarkably higher than that in the PBS group (Fig. 4B). The flow cytometry analysis results unveiled that the amount of cells in G0/G1 phase in the EVs-ALL group was significantly decreased (Fig. 4C), and the cell apoptotic rate was significantly lower (Fig. 4D) as well. As the transwell assay indicated, migration and invasion abilities were enhanced by EVs-ALL treatment (Fig. 4E, F). Our results showed that EVs from ALL patients’ blood could enter BALL-1 cells, and miRNA-181b-5p carried by the EVs could accelerate the tumorigenesis functions.

## Discussion

Overexpression and tumorigenesis roles of miRNA-181b-5 have been identified in gallbladder cancer and non-small cell lung cancer research [17, 18], thus we assumed that miRNA-181b-5p may serve as a tumor-promoting factor in ALL patients. Herein, we focused on ALL and studied the expression of miRNA-181b-5p and mechanism of it in peripheral blood-derived EVs of ALL patients.

Firstly, the high expression of miRNA-181b-5p was found in ALL patients through bioinformatics analysis. It was reported that miRNA-181a is significantly up-regulated in ALL and is associated with high probability of MRD and poor prognosis, and it can be used as a prognostic biomarker for child ALL [19]. The study conducted by Rita and others showed that silence of miRNA-181a-1/b-1 represses the progression of oncogene Notch1-induced T-ALL [20]. The up-regulated miRNA-181a-5p affects the WIF1 of ALL cells, indicating that miRNA-181a-5p-mediated Wnt signal may be part of the pathogenesis of ALL [21]. In addition, miRNA-181b-5p has been reported to promote tumor malignant progression by various pathways [17, 22, 23], while not been confirmed in ALL yet. These indicated that miRNA-181 might be incorporated in the malignant progression of ALL. To verify the function of miRNA-181b-5p in ALL cells, it was overexpressed in ALL cells. The results of cell assays unveiled that overexpressed miRNA-181b-5p remarkably enhanced the proliferation of ALL cells, decreased the amount of cells in G0/G1 phase and the apoptosis of ALL cells.

Additionally, miRNA-181b-5p is proved mainly present in serum exosomes and MVs. Therefore, here, EVs from peripheral blood of ALL patients and healthy people were extracted. Western blot was employed to test expression of EVs markers including CD63, CD9, TSG101, CD19 and CD40 and it was identified that EVs were successfully isolated. Further qRT-PCR results revealed that the expression of miRNA-181b-5p was significantly higher in EVs of ALL patients than that of healthy people. It is reported that miRNA-26a carried by exosomes derived from glioma stem cells can promote angiogenesis of glioma microvascular endothelial cells [24]. Mature T-cell leukemia/lymphoma cells can release EVs containing miRNA-21, miRNA-155 and vascular endothelial growth factor (VEGF), inducing NF-κB activation, resulting in changing of cellular morphology and increasing of proliferation, migration and angiogenic markers in recipient mesenchymal stem cells [25]. In this study, the extracted EVs were labeled with PKH67, and it was found that miRNA-181b-5p can be delivered into ALL cells by EVs. However, in this section, we did not entirely confirm the internalization process, which means the EVs may be attached to the surface of ALL cell membrane but not be absorbed into the cell. For this limitation, we are planning to arrange some more appropriate experiments to strengthen the logic of the study. Then, in order to prove that miRNA-181b-5p could enter ALL cells and affect the cell proliferation, cycle and apoptosis, MTT and flow cytometry were conducted for the EVs-ALL group. The results unveiled that cell proliferation was remarkably improved. These results suggest that EVs from peripheral blood of ALL patients can enter ALL cells and affect their proliferative capability. Interestingly, one research posted by Ying Wang identified a pro-angiogenetic role of EVs-derived miRNA-181b-5p in esophageal squamous carcinoma [23]. However, this effect was not examined in our study, so which we are planning an in-depth study on it in our next step.

In conclusion, our data demonstrate that miRNA-181b-5p carried by EVs from patients with ALL was capable of promoting the progression of ALL cells, affecting cell cycle and inhibiting the apoptosis of cancer cells. This may lay a good foundation for our future research regarding the influence of EVs on ALL.

## Data Availability

All the original data of this study are available from the corresponding author upon request.
